# Investigating women’s awareness and perceptions on human papillomavirus infection and oropharyngeal cancer in Italy

**DOI:** 10.3389/fpubh.2023.1195588

**Published:** 2023-08-28

**Authors:** Giovanna Paduano, Sara Vaienna, Giuseppe Maisto, Gabriella Di Giuseppe, Maria Pavia

**Affiliations:** Department of Experimental Medicine, University of Campania “Luigi Vanvitelli”, Naples, Italy

**Keywords:** awareness, human papillomavirus, oropharyngeal cancer, prevention, survey, vaccination, women

## Abstract

**Introduction:**

This study explored knowledge, attitudes and behaviors toward human papillomavirus (HPV) infection and oropharyngeal cancer (OPC) among women attending primary care services.

**Methods:**

The cross-sectional study was conducted from September to December 2022 in adult women attending three primary care services in Italy, who were invited to complete a self-administered questionnaire.

**Results:**

Overall, 34.7% of participants know that OPC is HPV-associated; knowledge was higher among women who had personal, familiar or friends’ experience of cancer, and who reported to often seek dental care, whereas it was lower in older women. The perception of risk of developing OPC was high for 26.4% of women and was higher in those who had experienced STD, who knew that early sexual debut is a risk factor for OPC, and who considered useful the role of dentists on the provision of information about OPC. Only 22.5% had received HPV vaccination, but 62% intended to receive it in the future. Moreover, 63.2% believe that HPV vaccination is very useful to prevent OPC and only 27% are concerned about health consequences of HPV vaccination.

**Conclusion:**

These findings indicate that women’s awareness of the role of HPV infection in the development of OPC is not satisfactory and underline the role that dentists might have in improving their patients’ awareness on HPV related OPC.

## 1. Introduction

It has been observed that the incidence of oropharyngeal squamous cell carcinoma (OPC) is rapidly increasing in several high-income countries (1, 2), eventually surpassing that of cervical cancer, which has already occurred in the UK and the United States (1, 2). Within this cancer site, HPV positive and HPV negative OPC are defined as separated entities with the HPV positive, which is mostly related to HPV 16 genotype, accounting for an increasing proportion of all cancers, and showing a more favorable prognosis (3). Generally, most HPV positive OPCs occur in men, whereas an increase in incidence has been observed among white women in the United States (4). In Italy, the incidence of HPV positive OPCs is lower compared to Northern Europe countries, but it has increased from 16.7% of all OPC in 2000–2006 to 46.1% in 2013–2018 (5). Moreover, it has been observed that the increase of HPV positive OPC is higher in females (5), and this trend has been confirmed by data from several cancer registries, which showed a differential gender related increase of HPV positive OPC (6).

The absence of a detectable precancerous lesion precludes the opportunity to establish screening programs enabling early diagnoses of OPC, as well as the use of such lesions for the evaluation of the efficacy of HPV vaccines in preventing OPC. There is however mounting evidence supporting the efficacy of HPV vaccines in the prevention of oral HPV infection (7–10), which has also been confirmed by two recent meta-analyses (11, 12). Moreover, from a different perspective, a study analyzing data from a registry has revealed that vaccinated subjects had lower risk of OPC compared to non-vaccinated (13).

Consequently, in June 2020, the US Food and Drug Administration has expanded the indication of the four and nine-valent HPV vaccines for the prevention of OPC (14), and the gender-neutral HPV vaccination strategies promoted by several countries, including Italy, appear to be an essential element of the prevention programs for OPC. However, before the beneficial effect of the vaccination strategies would be evident, since oral HPV infection is the primary risk factor for HPV-related OPC, and over 90% of oral HPV infections are sexually acquired (15), there is need to promote initiatives aimed at enhancing the awareness and perceptions of the public on the risks associated to HPV infections and their role in the development of OPC, as well as the adherence to HPV vaccination.

The assessment of knowledge, attitudes and behaviors on OPC, HPV infection and vaccination has been the objective of several surveys conducted in different populations, including dentists and dental hygienists (16–18), head and neck cancer health professionals (19), university (20) and dental students (21–26), as well as the adult population (27–30), but, since this cancer site has been traditionally associated to males, no studies have specifically explored women’s awareness on HPV related OPC.

Moreover, the only study that has specifically explored these issues in the adult population in Italy has involved patients attending dental practitioners (28) and this population may not easily be generalized to the Italian general population.

Considering the increasing incidence of HPV positive OPC in women in Italy, this study was conducted with the following objectives: (1) to explore women’s awareness on the association between HPV infection and OPC and related determinants, (2) to assess their attitudes toward the risk of developing OPC and related determinants, and (3) to measure HPV vaccination coverage in the eligible population and willingness to be vaccinated in non-vaccinated women.

## 2. Materials and methods

### 2.1. Study design

This investigation, which took place from September to December 2022 was designed as a cross-sectional survey-based study to be conducted on women attending primary care counseling services (PCCS). These services are peculiar to the Italian National Healthcare Service (NHS) and provide counseling for family planning and contraception, maternal care during pregnancy, counseling and treatment for sexually transmitted diseases (STDs), and primary and secondary prevention for cervical and breast cancers (31). Three PCCS were randomly selected in two provinces of Campania region, in the south of Italy, and approval and cooperation of the selected PCCS was sought through an invitation letter describing the project. A one-stage cluster sampling procedure was used to select the PCCS. From the list of the PCCS available from the Ministry of Health in 2 provinces in Campania region, (Naples and Caserta), southern Italy (32), three PCCS were selected by simple random sampling, and all adult women attending the selected PCCS at the time the research team visited the services were invited to participate to the survey. Women were approached by the study team in the waiting room or after they had completed the visit, were informed about the study objectives, that their participation was voluntary and that they could withdraw from the study at any time, and that confidentiality and anonymity were assured. Those who decided to participate were asked to sign a consent form to document their voluntary participation and invited to fill a standardized questionnaire which was designed in a self-administration format. Those who refused to participate were asked to respond to few socio-demographic questions and about reasons for non-participation.

Before study initiation, . the required minimum sample size was estimated using the following equation:

n $=\frac{Z_{1-\alpha ∕2}^{2}P\left(1-P\right)}{d^{2}}\times \mathrm{deff}\times \frac{1}{r}\;$ (33), where n is the sample size, $Z_{1-\alpha ∕2}^{2}$ is the statistics corresponding to the two sided confidence level, which was set at 95%, P is the expected prevalence of participating women who were able to recognize HPV as a possible cause of OPC, which, according to the literature, was set at 40% (20, 22, 27, 30), d is the precision, which was set at ±5%. This calculation yielded a minimum sample size of 369 subjects. However, since a cluster sampling strategy was used, this estimated sample was adjusted for the design effect (deff), which was set at 1.3, resulting in a minimum sample size of 480 subjects. Finally, the expected response rate (r) was set at 80% (27, 28, 34), which required to invite to participate 600 subjects. Moreover, to maximize the response rate, the research team visited the PCCS every 7 days to collect questionnaires and to offer the questionnaire to any as-yet non-participants. There were no incentives offered to women who accepted to participate in the survey.

### 2.2. Survey questionnaire

The questionnaire was prepared *ad hoc* and topics were selected through informal discussion within the research unit and comprehensive literature search. Based on an extensive literature review, the outline and the content of the questionnaire were developed from validated sources, used to investigate knowledge, attitudes and behaviors on HPV and OPC in dentists and dental hygienists (16, 18), university students (20–22, 34, 35) and adult population (27–30). Specifically, the questions to investigate the main sociodemographic and anamnestic characteristics were extrapolated from the study by Pelullo et al. (36), knowledge about the HPV infection from the studies by Pelullo et al. (35) and Nocini et al. (28), whereas attitudes toward HPV vaccination and behaviors related to the main preventive measures and sources of information were based on the studies by Mascaro et al. (34) and Pelullo et al. (35). To enable consistency and comprehensibility, the questionnaire was divided into five sections: the first explored socio-demographic and anamnestic characteristics (age, nationality, sexual orientation, marital status, working activity, number of persons in the household, number of children, smoking status, alcohol consumption, and personal or familiar history of cancer); the second investigated knowledge about HPV infections’ routes of transmission, risk factors and prevention as well as about HPV associated diseases, with specific attention to OPC. The third examined concern about developing HPV infection and OPC, and attitudes about effectiveness and safety of HPV vaccination; the fourth section explored participants’ behaviors in respect to sexual habits, HPV vaccination and PAP test adherence among eligible women, and reasons for not having undergone them, attendance to dental visits and information received by the dentist on HPV related OPC; finally, the fifth section was dedicated to sources of information on HPV infection and related diseases and the need of additional information. Each section elicited responses in a variety of formats: closed-ended questions with multiple answers possible, 10-point Likert scale options, yes or no answers and open-ended questions. Answering the questionnaire lasted an average of 15 min.

A pilot test on 50 women attending the PCCS was conducted to ensure question clarity, format and sequence, and refinements were made to improve flow and clarity. Specifically, in some questionnaires the final part was incomplete, and this was due to the length of the survey, therefore the sequence of the questions was rearranged and some of them were eliminated. Moreover, the format of some questions was modified from open to multiple choice answers to make it easier for the participant to respond.

The study was approved by the Ethics Committee of the University of Campania “Luigi Vanvitelli” (n 0022401/23.7.2021).

### 2.3. Statistical analysis

First, descriptive statistics, including frequencies, means, and SDs, were used to summarize retrieved information. Second, the chi-square test and the Student t-test were used to test the associations between several potential determinants and the outcomes of interest. Then, two stepwise multivariate logistic regression models with backward elimination were designed for the following outcomes: knowledge that OPC is an HPV infection related disease (Model 1), which was dichotomously recorded as 1 if the answer was “yes” and 0 if it was “no/do not know,” and perceived risk of developing OPC (Model 2), which was dichotomized as 1 if the answer was “high” (from 8 to 10 in the 10-point Likert scale) and 0 if it was “low” (from 1 to 7 in the 10-point Likert scale). The following independent variables were selected for both models: age group, years (ordinal) (1 = ≤29;2 = 30–39;3 = 40–49;4 = ≥50), marital status (0 = unmarried/widowed/separated/divorced, 1 = married/cohabitant), occupation (0 = unemployed, 1 = employed), smokers (0 = never, 1 = past/current), STD diagnosis(es) (0 = no,1 = yes), frequency of seeking dental care (0 = never/rarely, 1 = sometimes/often), perception of the usefulness of dentists in the provision of information about HPV infection (0 = not at all useful/not very useful, 1 = useful/very useful). In the first model, the following independent variables were also added: alcohol consumption (0 = never, once to four times a month, 1 = two or more times a week) and personal, family or friends’ history of cancer (0 = no, 1 = yes). In the second model knowledge that early sexual debut is a risk factor for OPC (0 = no/do not know, 1 = yes) was also added.

Models were developed according to the Hosmer and Lemeshow strategy (37) that includes the following steps: univariate analysis of each variable considered, using the appropriate test statistic; inclusion of any variable whose univariate test had a value of *p* ≤0.25; ways to include independent variables in the model (continuous, ordinal or categorical) took into account how each of these ways better fitted the data at the univariate analysis and we chose that way in the multivariate analysis. The independent variables that fitted these criteria or that were judged to potentially have an influence on the investigated outcomes were included in the appropriate model. Adjusted odds ratio (OR) and 95% confidence intervals (CI) were calculated. All reported 𝑝 values are two-tailed and a value ≤0.05 is considered statistically significant. The data were analyzed using Stata, version 15.1 (38).

## 3. Results

### 3.1. Study population

A total of 446 women out of 600 potential participants completed the survey yielding a response rate of 74.3%. Socio-demographic data and reason for attending the PCCS were similar in participating and non-participating women, and main reasons for non-participation were lack of time and lack of eyeglasses. Main reasons for attending the PCCS in participating women were provision of primary or secondary prevention procedures, such as vaccinations, mammography, pap smear or family planning counseling (32.6%); gynecological control visits (25.5%); gynecological problems (24.8%) or pregnancy periodical visits (14.8%). Table 1 displays an overview of women’s socio-demographic, anamnestic, and lifestyle characteristics. Women’s mean age was 39 years (SD ± 11.7), most were Italians (97.7%), more than half had sons/daughters (57.9%), and were married/cohabitant (54.3%), only 5.1% lived alone, 96.8% declared to be heterosexual, and 56.3% were employed. When asked about lifestyle characteristics, 30.9% declared to be current smokers and 18.4% that they consumed alcohol more than once a week; moreover, 4.9% mentioned a personal and 19.6% a family or friends’ history of cancer and, among these, 3% specifically of OPC, whereas 13.6% had ever had a diagnosis of an STD, mostly HPV infections (74.1%), followed by Chlamydia (7.4%) and genital herpes (5.6%).

**Table 1:** Respondents’ socio-demographic, anamnestic and lifestyle characteristics (*N* = 446).

Characteristics	*N*	%
Age group, years (446)^*^	39.05 ± 11.72 (16–69) **	
≤29	111	24.9
30–39	143	32.1
40–49	96	21.5
≥50	96	21.5
Nationality (444)^*^		
Foreigner	10	2.3
Italian	434	97.7
Sexual orientation (433)^*^		
Heterosexual	419	96.8
Homosexual	5	1.1
Bisexual	7	1.6
Other	2	0.5
Marital status (438)^*^		
Unmarried/widowed/separated/divorced	200	45.7
Married/ cohabitant	238	54.3
Number of cohabitants (433)^*^		
0	22	5.1
1	99	22.9
≥2	312	72
Son(s)/Daughter(s) (439)^*^		
No	185	42.1
Yes	254	57.9
Occupation (439)^*^		
Unemployed	192	43.7
Employed	247	56.3
Smokers (440)^*^		
Never	226	51.4
Past	78	17.7
Current	136	30.9
Alcohol consumption (440)^*^		
Never	124	28.2
Once a month	132	30
Two to four times a month	103	23.4
Two or three times a week	65	14.8
Four or more times a week	16	3.6
Personal history of cancer (445)^*^		
No	423	95.1
Yes	22	4.9
Family or friends history of cancer (434)^*^		
No	85	19.6
Yes	349	80.4
Sexually transmitted diseases (STD) diagnosis(es) (389)^*^		
No	336	86.4
Yes	53	13.6

*In brackets the number of respondents to each item.

**Mean ± Standard Deviation (Range).

### 3.2. Knowledge about HPV infection, OPC, and HPV vaccination

Results on knowledge are displayed in Table 2.

**Table 2:** Knowledge and attitudes on HPV infection and oropharyngeal cancer (OPC) (*N* = 446).

Knowledge	*N*	**%**
HPV is transmitted by sexual intercourse (417)^*^		
No	28	6.7
Yes	389	93.3
HPV infection may occur: (426)^*^		
Only in women	83	19.5
Only in men	6	1.4
In both sexes	290	68.1
Do not know	47	11
HPV infection is associated to:		
Cervical cancer (431)^*^ *(True)*	321	74.5
Genital warts (409)^*^ *(True)*	195	47.7
OPC (404)^*^ *(True)*	140	34.7
Penile cancer (405)^*^ *(True)*	123	30.5
Anal cancer (397)^*^ *(True)*	117	29.5
Urinary tract infections (413)^*^ (False)	160	38.7
Autoimmune diseases (398)^*^ (False)	78	19.6
Intestinal cancer (394)^*^ (False)	39	9.9
Lung cancer (396)^*^ (False)	30	7.6
Risk factors for HPV infection:		
Complete sexual intercourse (427)^*^ *(True)*	343	80.3
Incomplete sexual intercourse (418)^*^ *(True)*	219	52.4
Deep kiss (406)^*^ *(True)*	50	12.3
Needles exchange (407)^*^ (False)	110	27
Pregnancy (404)^*^ (False)	61	15.1
Oral contraceptives (402)^*^ (False)	36	8.9
Protective factors for HPV infection^a^:		
Condom use (420)^*^(True)	320	76.2
HPV vaccination (420)^*^ *(True)*	216	51.4
Delay of sexual debut (420)^*^ *(True)*	30	7.1
Pap-test (420)^*^ (False)	175	41.7
Vitamins (420)^*^ (False)	8	1.9
HPV vaccine is available in Italy (403)^*^		
No	97	24.1
Yes	306	75.9
HPV vaccination is available for both sexes (291)^*b^		
No	141	48.5
Yes	150	51.5
Risk factors for OPC:		
Smoking (412)^*^ *(True)*	220	53.4
Oral sex (412)^*^ *(True)*	197	47.8
No condom use (401)^*^ *(True)*	181	45.1
HIV infection (407)^*^ *(True)*	171	42
Multiple sex partners (403)^*^ *(True)*	144	35.7
Alcohol consumption (402)^*^ *(True)*	137	34.1
Early sexual debut (399)^*^ *(True)*	58	14.5
Attitudes		
Perceived risk of contracting HPV infection (420)^*^	4.67 ± 3.2 (1–10)^**^	
Low (from 1 to 7)	321	76.4
High (from 8 to 10)	99	23.6
Perceived risk of developing OPC (417)^*^	4.2 ± 3.2 (1–10)^**^	
Low (from 1 to 7)	307	73.6
High (from 8 to 10)	110	26.4
Belief that HPV vaccination is useful to prevent OPC (410) ^*^	7.63 ± 2.9 (1–10) ^**^	
Not useful (from 1 to 7)	151	36.8
Very useful (from 8 to 10)	259	63.2
Concern that HPV vaccination may have serious consequences on health (408)^*^	5.1 ± 3.14 (1–10)^**^	
Not worried (from 1 to 7)	298	73
Very worried (from 8 to 10)	110	27

* In brackets the number of respondents to each item.

^a^ Multiple responses allowed.

^b^ Only for those who reported that HPV vaccine is available in Italy.

** Mean ± Standard Deviation (Range).

Most participants were aware that HPV infection is sexually transmitted (93.3%), but only two thirds (68.1%) that it is an infection that may occur in both sexes.

Knowledge of HPV associated diseases was higher for cervical cancer (74.5%), followed by genital warts (47.7%), OPC (34.7%), penile (30.5%), and anal (29.5%) cancer, whereas 38.7% and 19.6% of respondents erroneously indicated urinary tract infections and autoimmune diseases as HPV related, respectively.

Among risk factors for HPV infections, 80.3% indicated complete sexual intercourse, 52.4% incomplete sexual intercourse, 12.3% deep kisses, 27% needles exchange and 15.1% pregnancy. Moreover, only 76.2% and 51.4% acknowledged condom use and HPV vaccination as protective factors for HPV infection, respectively, and only 7.1% the delay of sexual debut.

Poor knowledge on HPV vaccination was confirmed by the response on availability of HPV vaccination for both sexes, which was indicated by only 51.5% of respondents.

Smoking was identified as a risk factor for OPC by 53.4% of respondents, followed by oral sex (47.8%), unprotected sex (45.1%), HIV infection (42%), multiple sex partners (35.7%), and alcohol consumption (34.1%), whereas only 14.5% indicated early sexual debut.

### 3.3. Attitudes toward HPV infection, OPC, and HPV vaccination

Table 2 shows respondents’ attitudes toward HPV infection, OPC and HPV vaccination. Only 23.6% of respondents perceived to be at high risk of contracting HPV infection, and a similar proportion (26.4%) of developing OPC.

Belief that HPV vaccination is effective in the prevention of OPC is expressed by 63.2% of participants, and only 27% are worried by possible side effects of HPV vaccination. When women who had not been vaccinated against HPV were asked about their willingness to receive that vaccination in the future 62% expressed their intention to get vaccinated, and the main reasons were to reduce their risk of getting HPV infection (80%), the feeling that it is a useful vaccination (43.4%), its effectiveness in cancer prevention (39.8%), and the recommendation by a physician (21.4%). On the contrary, among those who stated that they would not be willing to uptake HPV vaccination, the most common reasons were the low perceived risk of HPV infection (61.7%), concerns about the safety of the vaccine (24.3%), the belief that the vaccine is not useful (5.6%) and having been advised against the HPV vaccination (5.6%).

### 3.4. Behaviors related to HPV infection and OPC prevention

Table 3 reports the results on respondents’ sexual activity as well as their participation to prevention activities related to HPV infections and related diseases. Only 1.4% of the women had never had a sexual intercourse in their lifetime. Among the sexually experienced, more than half (56.3%) were less than 18 at their sexual debut, and only 4.5% did not have any sexual partner in the previous year, although most of them (84%) reported only one partner in the previous year. Oral sex was reported by 85.9% of respondents, and by 12.1% with more than one partner in the previous year.

**Table 3:** Behaviors related to prevention of HPV infection and associated diseases (*N* = 446).

Behaviors	*N*	%
Experience of sexual intercourse (426)^*^		
No	6	1.4
Yes	420	98.6
Age of sexual debut (382)^*+^	17.59 ± 2.66 (12–36)^ò^	
≤17	215	56.3
18–23	154	40.3
≥24	13	3.4
Sexual intercourse, previous year (401)^*+^		
No	18	4.5
Female	10	2.5
Male	372	92.7
Both sexes	1	0.3
Number of sexual partners, previous year (368)^*#^		
>1	59	16
1	309	84
Oral sex, lifetime (390)^*+^		
No	55	14.1
Yes	335	85.9
Oral sex partners, previous year (331)^*a^		
0	7	2.1
1	284	85.8
>1	40	12.1
Having undergone HPV vaccination (258)^*b^		
No	200	77.5
Yes	58	22.5
Intent to receive HPV vaccine in the future (329)^*°^		
No	125	38
Yes	204	62
Having ever undergone a Pap-test (361)^*c^		
No	41	11.4
Yes	320	88.6
Frequency of seeking dental care: (413)^*^		
Never/rarely	195	47.2
Sometimes/often	218	52.8
Having been informed about HPV and oral cancer by a dentist (413)^*^		
No	385	93.2
Yes	28	6.8
Perception of the usefulness of information about HPV received by a dentist (28)^*d^		
Not at all useful/not very useful	5	17.9
Useful/very useful	23	82.1
Perception of the usefulness of information about OPC received by a dentist (28)^*d^		
Not at all useful/ not very useful	5	17.9
Useful/very useful	23	82.1

^*^In brackets the number of respondents and eligible to the question.

^ò^Mean ± Standard Deviation (Range).

^+^Among those who reported sexual intercourse.

^#^Among those who reported sexual intercourse in the previous year.

^a^Among those who reported oral sex.

^b^ Only in age-eligible women (aged < 26 years from 2007 to the present).

^c^Only in age-elegible women (aged ≥ 25 years).

°Among those who had not received HPV vaccination.

^d^Only for those who answered “Yes” at the question “Having been informed about HPV and oral cancer by a dentist”.

Within eligible women, only 22.5% had been vaccinated against HPV infection, 88.6% had ever undergone a Pap-test and, of these, 93.5% in the previous 3 years. The most frequent reasons for not under going Pap-test were fear of results (68.4%) and having been advised against it (26.3%).

Among women who reported to having been visited by a dentist, only 6.8% reported to have been informed about HPV infection risk factors and OPC, and 82.1% considered very useful the information on HPV and OPC received by the dentist.

### 3.5. Sources of information

A minority of women had participated to information/education activities on HPV infection and related diseases as well as on other STDs (9.9%), whereas a large majority expressed their interest in receiving further information provided by physicians on HPV infection (82.1%) and HPV related OPC (84.8%).

### 3.6. Multivariate analysis

Results of the multivariate logistic regression are shown in Table 4. Knowledge that OPC is an HPV infection associated disease was significantly higher in women who had personal, familiar or friends’ experience of cancer (OR = 1.97, 95%CI = 1.05–3.69), and who reported to often seek dental care (OR = 1.82, 95%CI = 1.13–2.93), whereas it was lower in older women (OR = 0.75, 95%CI = 0.60–0.94), and in past or current smokers (OR = 0.62, 95%CI = 0.39–1.00), although in this case the association was slightly statistically significant (Model 1 in Table 4).

**Table 4:** Multiple logistic regression analysis about knowledge of the link between HPV infection and OPC (Model 1) and high perceived risk of developing OPC (Model 2) according to several explanatory variables.

Variable	OR		95% CI	*p*
°Model 1. Knowledge that OPC is an HPV infection related disease.				
Log likelihood = −198.19, χ^2^ = 19.28 (4 df), *p* = 0.0007, No. of obs = 323				
Age group, years (ordinal) (1 = ≤29;2 = 30–39;3 = 40–49;4 = ≥50)	0.75		0.60–0.94	0.012
Frequency of seeking dental care				
Never/rarely	1.00*			
Sometimes/often	1.82		1.13–2.93	0.015
Personal, family or friends’ history of cancer				
No	1.00*			
Yes	1.97		1.05–3.69	0.034
Smokers				
Never	1.00*			
Past/current	0.62		0.39–1.00	0.052
aModel 2. High perceived risk of developing OPC.				
Log likelihood = −141.19, χ2 = 24.52 (6 df), p = 0.0004 No. of obs =270				
Sexually transmitted diseases (STD) diagnosis (es)				
No	1.00*			
Yes	2.36		1.14–4.91	0.021
Early sexual debut is a risk factor for OPC				
No/do not know	1.00*			
Yes	2.29		1.07–4.90	0.033
Frequency of seeking dental care				
Never/rarely	1.00*			
Sometimes/often	0.43		0.24–0.77	0.005
Marital status				
Unmarried/widowed/separated/divorced	1.00*			
Married/cohabitant	0.74		0.41–1.34	0.322
Smokers				
Never	1.00*			
Past/current	1.42		0.79–2.55	0.245
Perception of the usefulness of dentists in the provision of information about OPC				
Not at all useful/not very useful	1.00*			
Useful/very useful	1.69		0.91–3.14	0.094

*Reference category.

°The following variables were deleted by the backward elimination procedure: marital status, occupation, alcohol consumption, STD diagnosis(es), perception of the usefulness of dentists in the provision of information about HPV infection, perception of the usefulness of dentists in the provision of information about OPC.

a The following variables were deleted by the backward elimination procedure: age group, years (ordinal), occupation, perception of the usefulness of dentists in the provision of information about HPV infection.

When exploring attitudes, the perception of risk of developing OPC was significantly higher in those who were aware that early sexual debut is a risk factor for OPC (OR = 2.29, 95%CI = 1.07–4.90), and who considered useful the role of dentists in the provision of information about OPC, although not significantly (OR = 1.69, 95%CI = 0.91–3.14); conversely this perception of risk was significantly lower in those who declared higher frequency of dental care seeking (OR = 0.43, 95%CI = 0.24–0.77) (Model 2 in Table 4).

## 4. Discussion

As far as we know this is the only study exploring awareness and attitudes on the relationship between HPV infection and OPC that has been specifically focused on women, a group of the population that has not been given enough attention, despite there has been evidence of an increasing incidence of OPC affecting women.

This study revealed that women’s awareness on the association between HPV infection and OPC is still lacking, with only 34.7% recognizing this link, compared to 74.5% who indicated cervical cancer, but this frequency is higher than that reported by Verhees et al. (27) (29.2%) and Dodd et al. (20) (26.7%), and similar to that indicated by Williams et al. (30) (36%) and Lechner et al. (39) (38.7%) in the general population; indeed a systematic review and meta-analysis has shown that knowledge of HPV-associated OPC in the general population ranged from 7 to 57% (40), suggesting that interventions to increase awareness of HPV association with non-cervical cancers should be considered.

Smoking has been traditionally associated to the development of OPC, and it was in this study the mostly frequently mentioned risk factor (53.4%), but, surprisingly, it was followed by oral sex (47.8%), whereas alcohol consumption was recognized by only 34.1%. This finding is interesting and suggests that the sexually transmitted origin of this cancer has begun to spread in the general population and specifically in women.

It is remarkable that frequent seeking of dental care was one of the determinants of knowledge of the association between HPV and OPC, and that most women considered very useful the information on HPV and OPC provided by dentists. Conversely, it is concerning that only few respondents (6.8%) had been informed by dentists on these topics. This is not surprising, since similar results have been reported by Nocini et al. (28) (11%), and surveys investigating dentists’ behaviors have shown that most declared they do not discuss about HPV-related cancer prevention (17), as well as about the connections between HPV and OPC with their patients (41). There is plenty of literature demonstrating the significant role played by health professionals in motivating the public to undergo prevention and healthcare activities (36, 42, 43), and it is concerning that dentists do not engage in the advice of their patients about the connection between HPV infection and OPC; however, it has been reported that dentists lack the skills to effectively communicate and educate their patients on these topics without embarrassment, and that appropriate training would improve communication skills and ultimately expand patients’ education on HPV-related OPC (17). Moreover, the awareness that OPC may be HPV related was age dependent, with younger women showing higher knowledge; this result is in line with previous studies in the general population in Germany (44) and in the Netherlands (27), and it is probably related with younger subjects’ more frequent experience of HPV infection and with their eligibility for HPV vaccination. The finding that a family or personal history of cancer was a predictor of higher awareness on the association of HPV infection and OPC has been reported for risk factors of other cancers, including breast cancer (36), but did not significantly influence knowledge about the HPV infection and OPC in the study by Nocini et al. (28).

HPV vaccination has proven to be very effective in the prevention of cervical cancer, and there is growing literature showing that it may be effective in the prevention of oral HPV infection (7–12). It is, therefore, noteworthy that almost two thirds of the participant women believe that HPV vaccination is effective in the prevention of OPC, considering that HPV vaccination coverage in the eligible population in Italy is far from reaching the recommended thresholds. A low HPV vaccination coverage is confirmed by the results of this study, since only 22.5% of the eligible women reported to have undergone HPV vaccination. It is promising, however, that among non-vaccinated women, 62% expressed their willingness to undergo, although this finding is slightly lower than that found in a study in young adult women in the same area (71%) (34), but similar to that of nursing student in the same area (65.3%) (35), and higher that that reported in women in Slovenia (49.6%) (45). Indeed, it is known that intention to undergo vaccination may not always predict real vaccination uptake (46), and identification of determinants of hesitancy is a key component in the design of interventions aimed at the achievement of successful vaccination coverage. It has been reported that predisposing factors, including willingness, may be less relevant compared to enabling factors (47), indicating the need to implement innovative strategies to reach and catch-up women who had not the opportunity to be vaccinated.

The risk of developing OPC is perceived to be high by a quarter of the participant women and the finding that this perception is significantly higher in women who had been diagnosed STDs and know that early sexual debut is a risk factor for OPC again testifies that knowledge and perception that OPC may be related to sexual transmission is spreading in women’s general population.

The findings of this study on reported risky and preventive behaviors for OPC are concerning. Indeed, smoking was declared by 30.9%, and, similarly to other studies (20, 48, 49), oral sex was experienced by a large majority (85.9%), with 12.1% reporting more than one oral sex partner in the previous year. These results, coupled with a low adherence to preventive measures, such as HPV vaccination (22.5%), demonstrate the potentials for expecting high incidence of OPC in women, in the absence of interventions aimed at increasing awareness on this issue.

Results from this study are valuable to expand knowledge on the awareness and beliefs on the role of HPV infection in the development of OPC in the general population of women but have to be interpreted in the light of some limitations. First of all, the study was cross-sectional, not allowing to draw conclusions on causality about the observed associations; moreover, reporting on sensitive topics such as sexual experiences, may have been biased by “socially desirable” responses. However, confidentiality and anonymity were assured to participants, mitigating the impact of this issue on the results. Third, women were recruited in the PCCS, and therefore may not be completely representative of the general population of the same area, although it may be hypothesized that it is mostly representative of the women in childbearing age. Finally, the survey was carried out only in one geographic area of Italy, therefore, caution on generalizability of results is due. Besides these limitations, this survey was well-designed and had a considerable response rate (74.3%), which supports validity of the estimates.

The results of this survey indicate that women’s awareness and perceptions on the role of HPV infection in the development of OPC are far from being satisfactory, and this concern is worsened by the low HPV vaccination coverage in the eligible population. Moreover, the study finding revealing the role that dentists might have in improving their patients’ awareness indicate that interventions might also be targeted to dentists, to improve their skills in communication on HPV related cancers. This might help to also increase HPV vaccination uptake, and ultimately reduce the emerging increase of OPC in women.

## Data availability statement

The raw data supporting the conclusions of this article will be made available by the authors, without undue reservation.

## Ethics statement

The studies involving human participants were reviewed and approved by Ethics Committee of the University of Campania “Luigi Vanvitelli”. Written informed consent to participate in this study was provided by the participants.

## Author contributions

GP and GG participated in the conception and design of the study, interpretation and wrote section of the manuscript. GP, SV, and GM contributed to the data collection and data analysis. MP designed the study, was responsible for the statistical analysis and interpretation, drafted and wrote the article. All authors contributed to the article and approved the submitted version.

## Conflict of interest

The authors declare that the research was conducted in the absence of any commercial or financial relationships that could be construed as a potential conflict of interest.

## Publisher’s note

All claims expressed in this article are solely those of the authors and do not necessarily represent those of their affiliated organizations, or those of the publisher, the editors and the reviewers. Any product that may be evaluated in this article, or claim that may be made by its manufacturer, is not guaranteed or endorsed by the publisher.

## Author disclaimer

Preliminary results have been presented at the 3rd Edition of the “Giornate della ricerca scientifica e delle esperienze professionali dei giovani” of the Italian Public Health Association (SItI), March 25–26, 2022.
